# Impact of open lung ventilation on right ventricular outflow impedance assessed by transoesophageal echocardiography

**DOI:** 10.1186/cc9592

**Published:** 2011-03-11

**Authors:** S Salah, H El-Akabawy

**Affiliations:** 1 Cairo University, Cairo, Egypt

## Introduction

Open lung concept ventilation is a method of ventilation intended to maintain end-expiratory lung volume by increased airway pressure [1]. Since this could increase right ventricular (RV) afterload, we investigated the effect of this method on RV outflow impedance during inspiration and expiration using transoesophageal echo-Doppler in a trial to differentiate the RV consequence of increasing lung volume from those secondary to increasing airway pressure during mechanical ventilation.

## Methods

Thirty stable patients on MV because of different causes were enrolled prospectively in this single-center, cross-sectional clinical study. Each patient was firstly subjected to conventional ventilation (CV) with volume-controlled ventilation, followed by OLC ventilation by switching to a pressure-controlled mode, then a recruitment maneuver applied until PaO_2_/FiO_2 _> 375 torr. Hemodynamic (MAP, CVP and HR) and respiratory (peak, plateau and mean airway pressure and total and dynamic lung compliance) measurements were recorded before, 20 minutes after a steady state of both CV and 20 OLC ventilation. Also, transoesophageal ECHO Doppler was performed at the end of inspiration and end of expiration to calculate the mean acceleration (AC_mean_), as a marker of the RV outflow impedance, 20 minutes after a steady state of both CV and OLC ventilation.

## Results

During inspiration, AC_mean _was significantly lower during CV compared with OLC ventilation (*P *< 0.001). Inspiration did not cause a significant decrease in AC_mean _compared with expiration during OLV (*P *< 0.001) but did do so during CV. In comparison with baseline and CV, OLC ventilation was associated with a statistically significant higher CVP (*P *< 0.001 for both), higher total quasi-static lung compliance (*P *< 0.001 for both) and dynamic lung compliance (*P *= 0.001 for both). Moreover, the PaO_2_/FiO_2 _ratio of OLV was significantly higher than in baseline and CV (*P *< 0.001 for both).

## Conclusions

OLC ventilation does not change RV afterload during inspiration and expiration as RV afterload appears primarily mediated through the tidal volume. Moreover, OLC ventilation provides a more stable hemodynamic condition and better oxygenation and lung dynamics.
